# Case Report: Concurrent maternal uniparental disomy-type Prader-Willi syndrome and X-linked G6PD deficiency—an area of focus on multi-gene screening for imprinting disorders

**DOI:** 10.3389/fped.2026.1924208

**Published:** 2026-10-14

**Authors:** Qiong Zhou, Jia-Ming Yuan, Chao-Chun Zou

**Affiliations:** 1 Department of Endocrinology and Metabolism, Hangzhou Children’s Hospital, Hangzhou, China; 2 Department of Endocrinology, Children’s Hospital, Zhejiang University School of Medicine, National Clinical Research Center for Child Health, Hangzhou, China

**Keywords:** Prader-Willi syndrome, glucose-6-phosphate dehydrogenase deficiency, uniparental disomy, comorbid genetic disorders, X-linked hereditary disease

## Abstract

**Background:**

Prader-Willi syndrome (PWS) is a rare imprinting disorder caused by functional impairment of imprinted genes within the paternal 15q11.2-q13 region. Glucose-6-phosphate dehydrogenase (G6PD) deficiency is an X-linked hereditary disorder. Cases describing the coexistence of maternal uniparental disomy (UPD)-type PWS and G6PD deficiency are extremely scarce in global clinical reports. This article summarizes three male infant cases from our center and further sorts out previously published literature on PWS combined with other genetic disorders to analyze shared genetic characteristics and clinical phenotypes of such combined genetic conditions.

**Methods:**

We performed retrospective sorting and analysis on three male infants simultaneously diagnosed with maternal UPD-type PWS and G6PD deficiency between August 2022 and December 2024 at the Children's Hospital, Zhejiang University School of Medicine. Meanwhile, systematic retrieval of published literature from 1956 to 2025 was carried out across PubMed, Scopus Preview, Cochrane Reviews, Ovid and Semantic Scholar, and 28 documented PWS patients complicated by other single-gene or chromosomal genetic disorders were collated for comparative analysis. Genetic variant pathogenicity was judged against the American College of Medical Genetics and Genomics (ACMG) genetic variant classification criteria.

**Results:**

All three enrolled infants presented typical core manifestations of PWS, including neonatal hypotonia, feeding difficulties, and cryptorchidism, without jaundice, anemia, dark urine or other G6PD-related symptoms. Genetic testing confirmed maternally inherited hemizygous G6PD variants: one case carried c.152C >T classified as likely pathogenic, and two carried c.1388G >A classified as pathogenic. Literature collation identified 14 PWS patients complicated with sex chromosome abnormalities and 15 patients with autosomal chromosomal/genomic or monogenic disorders.

**Conclusions:**

To our knowledge, this is the first case series reporting maternal UPD-type PWS combined with X-linked G6PD deficiency; it supplements novel clinical observations of this rare dual diagnosis and expands the recognized spectrum of PWS comorbid genetic disorders. For maternal UPD-PWS patients, expanded trio-whole exome sequencing (trio-WES) or trio-whole-genome sequencing (trio-WGS) is worth clinical consideration even without atypical phenotypes, to screen for hidden maternal multi-locus pathogenic variants. Given the small sample size of only three patients, additional multi-center data are needed to validate the clinical and genetic patterns of this combined phenotype. Keywords: Prader-Willi syndrome; Glucose-6-phosphate dehydrogenase deficiency; Uniparental disomy; Comorbid genetic disorders; X-linked hereditary disease

## Background

Prader-Willi syndrome (PWS) is a rare multi-system genetic disorder induced by functional abnormalities of imprinted genes located on paternal 15q11.2-q13 (1, 2). Its overall population incidence is estimated at 1 in 10,000 to 1 in 30,000 (3). PWS displays distinct stage-specific clinical manifestations: generalized hypotonia and severe feeding impairment dominate infancy, which progress to uncontrollable hyperphagia and progressive obesity in early childhood (4). Most affected individuals present with global developmental delay, variable cognitive deficits, and behavioral disturbances (4). Endocrine phenotypes frequently consist of gonadal dysgenesis, hypogonadism and short stature (5). Additional clinical manifestations include characteristic craniofacial features, sleep-disordered breathing, scoliosis, hip malformations and multiple obesity-associated complications (5). Four major molecular subtypes of PWS have been established by clinical consensus: paternal deletion of 15q11.2-q13.1 (65%–75% of cases), maternal uniparental disomy (UPD, 20%–30%), imprinting center microdeletion or point mutation (1%–3%), and rare balanced translocations involving the 15q region (<1%) (2, 5). Multiple clinical reports have documented PWS co- occurring with independent genetic conditions, including Klinefelter syndrome (6–8), DiGeorge syndrome (9), and autosomal recessive congenital ichthyosis caused by homozygous CERS3 variants (10, 11). Glucose-6-phosphate dehydrogenase (G6PD) deficiency arises from pathogenic variants in the X- linked G6PD gene, resulting in reduced or absent enzyme catalytic activity (12). The disorder predominantly affects hemizygous males, with an estimated global affected population exceeding 400 million (12). Notably, most variant carriers remain lifelong asymptomatic without spontaneous hemolytic episodes. At present, published clinical reports describing concurrent maternal UPD-type PWS and G6PD deficiency are nearly absent worldwide. This study analyzes three such infants in our cohort and synthesizes existing literature to clarify clinical and genetic features of PWS with concomitant genetic disorders, providing a diagnostic reference for pediatric genetic clinicians.

## Case presentation

### Study design and ethical approval

This retrospective clinical study collected clinical and molecular data of children with dual diagnoses of maternal UPD-PWS and G6PD deficiency. The research protocol obtained ethical clearance from the Ethics Committee of Children's Hospital, Zhejiang University School of Medicine (approval number: 2019-IRB-025). Written informed consent was acquired from all legal guardians prior to genetic testing and data collection, following the Declaration of Helsinki and institutional research guidelines.

### Patient baseline information and clinical manifestations

Three male Han infants conceived without consanguinity were enrolled; parental childbearing ages ranged from 27 to 30 years, and no family history of hereditary disease was reported by any household. Full clinical indicators are summarized in Table 1. Patient 1: Current age 2 years and 2 months, diagnosed at 2 months old; full-term cesarean delivery, birth weight 2.45 kg. The mother developed gestational diabetes during gestation and reported reduced fetal movement. The infant exhibited severe neonatal hypotonia and feeding failure requiring neonatal hospitalization; nasogastric tube feeding was not needed. Sticky saliva and patent foramen ovale (PFO) were identified on postnatal evaluation. No typical PWS facial dysmorphism was observed. Serial laboratory tests showed no jaundice, anemia or dark urine; liver, renal, thyroid and glycemic indices remained within normal limits during follow-up. Patient 2: Current age 2 years and 5 months, diagnosed at 1 month old; full-term cesarean delivery, birth weight 2.7 kg. Prenatal maternal mild anemia and decreased fetal movement were recorded. Classic PWS facial gestalt, generalized hypopigmentation and sticky saliva were noted; echocardiography confirmed PFO at birth. Nasogastric feeding was required in the neonatal period, and hip dysplasia was detected on follow-up imaging in August 2023. No hemolytic signs consistent with G6PD deficiency were identified across serial assessments. Patient 3: Current age 2 years old, diagnosed at 2 months old; preterm cesarean delivery at 37 weeks gestation, birth weight 2.2 kg. The mother had mild anemia without reduced fetal movement during pregnancy. Neonatal hypotonia and poor feeding necessitated prolonged nasogastric tube support. No sticky saliva, cardiac malformations or skeletal abnormalities were detected. Routine hematological screening revealed no evidence of G6PD-related hemolysis throughout observation. Shared clinical phenotypes across all three patients included cryptorchidism, blunted pain perception, inability to hold the head upright by 4 months of age, and persistent failure to thrive in early infancy. All children completed continuous follow-up exceeding 1 year, with no documented hemolytic crises or other acute complications related to G6PD deficiency. Of note, several cardinal PWS features, including hyperphagia and scoliosis, are age-dependent manifestations that typically emerge later in childhood; given the young age of the present patients (2–2.5 years), these features were not yet fully assessable and are therefore indicated as “not yet evaluable (age-dependent)” in Table 1; progressive obesity, hip dislocation and other late-onset features will be evaluated during ongoing longitudinal follow-up.

**Table 1:** Clinical characteristics of 3 children.

Clinical features	Patient 1	Patient 2	Patient 3
Population characteristics			
Gender	Male	Male	Male
Age	2 years 2 months old	2 years 5 months old	2 years old
Age at diagnosed	2 months old	1 month old	2 months old
Gestational age (weeks)	40	41	37
Mode of delivery	Cesarean	Cesarean	Cesarean
Birth weight (kilograms)	2.45	2.7	2.2
Age of mother at childbirth	27 years old	28 years old	27 years old
Age of father at childbirth	29 years old	27 years old	30 years old
Prader–Willi syndrome-related			
Genotype	UPD(15)mat	Mixed UPiD & UPhD(15)mat	UPD(15)mat
Hypotonia in the neonatal period	+	+	+
Feeding difficulties in infancy	+	+	+
Nasal tube feeding	−	+	+
Neonatal hospitalization	+	+	+
Slow weight gain in early infancy	+	+	+
Characteristic facial features	−	+	−
Hypogonadism	+	+	+
Growth retardation	+	+	+
Hyperphagia ^a^	Not yet evaluable (age-dependent)	Not yet evaluable (age-dependent)	Not yet evaluable (age-dependent)
Decreased fetal movement	+	+	−
Sleep disorders/apnea	−	−	−
Short stature	−	−	−
Hypopigmentation	−	+	−
Eye abnormalities	−	−	−
Sticky saliva	+	+	−
Pruritus	−	−	−
Unstable body temperature	−	−	−
Scoliosis ^a^	Not yet evaluable (age-dependent)	Not yet evaluable (age-dependent)	Not yet evaluable (age-dependent)
Hip problems	−	+	−
G6PD deficiency-related			
Genotype	c.152C >T (p.Thr51Ile)	c.1388G > A (p.Arg463His)	c.1388G > A (p.Arg463His)
Classification (ACMG)	LP (PM1, PM2, PP2, PP3)	P (PM1, PM5, PS3, PS4)	P (PM1, PM5, PS3, PS4)
Inheritance	Hemizygote	Hemizygote	Hemizygote
Jaundice	−	−	−
Anemic	−	−	−
Urine color deepens	−	−	−
Other			
Heart problems	PFO	PFO	−
Kidney problems	−	−	−
Hypothyroidism	−	−	−
Convulsions	−	−	−

G6PD, glucose-6-phosphate dehydrogenase; LP, likely pathogenic; PFO, patent foramen ovale; –, absent; +, present.

a Age-dependent feature not yet evaluable at the patients’ current ages (2–2.5 years); will be evaluated during ongoing longitudinal follow-up.

### Disease timeline summary

Prenatal period: Two mothers reported diminished fetal movement; one mother had gestational diabetes mellitus, while two experienced mild anemia during gestation. Neonatal period: All infants presented severe hypotonia and feeding dysfunction requiring neonatal ward admission; two patients were diagnosed with patent foramen ovale via echocardiography. 1–2 months postnatal: Clinical suspicion of PWS prompted comprehensive molecular genetic testing. 2 months to 2 years of age: Combined molecular assays confirmed maternal UPD-type PWS plus pathogenic G6PD variants; regular multi-system follow-up was conducted without acute adverse events attributable to G6PD deficiency.

### Auxiliary and genetic diagnostic methods

Routine auxiliary examinations: Blood biochemistry, thyroid function, glycemic markers, cardiac/abdominal/gonadal ultrasound, and radiological imaging of the skull, hips and spine were performed for every child. Genetic testing panel: Methylation-specific multiplex ligation-dependent probe amplification (MS- MLPA), short tandem repeat (STR) analysis and trio-whole exome sequencing (trio-WES) were implemented collaboratively. Pathogenicity classification of all detected variants followed ACMG standardized guidelines (13). PWS molecular diagnosis basis: MS-MLPA identified aberrant methylation signals at 15q11.2-q13 without copy number variation in all three patients. Combined STR and trio-WES results confirmed maternal uniparental disomy of chromosome 15; Patient 2 harbored mixed maternal uniparental isodisomy (UPiD) and heterodisomy (UPhD), while Patients 1 and 3 carried simple maternal UPD affecting the 15q imprinted region.

### G6PD variant molecular diagnosis basis

Patient 1: Hemizygous missense variant c.152C >T (p.T51I), maternally inherited. This variant is rarely recorded in the GnomAD population database; multiple functional prediction algorithms (SIFT, PolyPhen, MutationTaster) marked it as highly damaging. ClinVar designated it likely pathogenic, LOVD and HGMD classified it as disease-causing mutation (DM), satisfying ACMG criteria PM1, PM2, PP2, PP3. Patients 2 and 3: Hemizygous missense variant c.1388G >A (p.Arg463His), maternally inherited. This locus represents a recognized G6PD mutation hotspot with a population allele frequency of 0.0071724. ClinVar entries confirm pathogenicity, and functional validation studies verify impaired protein function; predictive tools including REVEL, SIFT, PolyPhen and MutationTaster classify the variant as damaging, meeting ACMG pathogenic standards PM1, PM5, PS3, PS4.

### Clinical intervention and follow-up outcome

All patients received standardized multi-disciplinary PWS management, including targeted nutritional support and feeding rehabilitation for infantile feeding dysfunction, plus regular endocrine surveillance for cryptorchidism and gonadal maturation. For concomitant G6PD deficiency, guardians received standardized counseling to strictly avoid fava beans and oxidative hemolytic-triggering medications. No targeted pharmacotherapy was administered for G6PD deficiency given the complete absence of related clinical manifestations. After over one year of continuous follow-up, all children maintained normal liver function, renal function, myocardial enzymes, thyroid function and routine blood counts. No spontaneous hemolysis, severe infection or other G6PD-associated adverse events were observed. Core PWS phenotypes including hypotonia and developmental delay persisted as expected, without new-onset organ complications.

### Literature review

We systematically retrieved PubMed, Scopus Preview, Cochrane Reviews, Ovid and Semantic Scholar for publications spanning 1956–2025 using search terms “Prader–Willi syndrome”, “comorbid genetic disorders”, “concurrent monogenic disease”, yielding an initial pool of 4,908 citations. After filtering for complete dual molecular diagnoses, 28 well-documented PWS patients with concomitant independent genetic disorders were categorized into two subgroups: sex chromosomal abnormalities (14 cases) and autosomal genetic disorders (15 cases). The sum of the two subgroups (29) exceeds the stated total of 28 unique patients, because the patient with both isochromosome Xq and Tay-Sachs disease (14) was included in both subgroups; the stated total of 28 unique patients is therefore correct. PWS combined with sex chromosome abnormalities (14 cases): Documented conditions include Klinefelter syndrome, trisomy X, 47,XYY syndrome and mosaic Turner syndrome (6–8, 15–23). Both paternal deletion and maternal UPD PWS subtypes were equally represented (8 vs. 6 cases). Literature records span nearly seven decades (1956–2025), yet only four reports clarified the parental origin of the aberrant sex chromosomes (7, 15, 16). PWS combined with autosomal genetic disorders (15 cases): In accordance with the underlying genetic mechanism, these cases were classified into (i) autosomal chromosomal/genomic disorders, including Down syndrome (trisomy 21) (24), DiGeorge syndrome (22q11.2 deletion syndrome) (9), and Sotos syndrome (NSD1 deletion) (25); and (ii) monogenic disorders, which were further subdivided into autosomal recessive disorders unmasked by maternal UPD of chromosome 15 (Bloom syndrome (26), Tay-Sachs disease (14, 27), autosomal recessive congenital ichthyosis (10, 11), hereditary spastic paraplegia type 11 (28), isovaleric acidemia (29), CAPN3-associated calpainopathy (30), and deafness–infertility syndrome due to STRC/CATSPER2 contiguous gene deletion (31)) and other monogenic disorders unrelated to chromosome 15 UPD (Pitt–Hopkins syndrome (32), ATP8A2-related cerebellar ataxia (33)). The majority of autosomal recessive comorbidities arose in patients with maternal UPD-type PWS, in whom full-chromosome 15 maternal disomy enables homozygous expression of recessive variants transmitted from the mother (34). This mechanism-based classification better reflects the distinct genetic pathways underlying PWS comorbidity and is adopted in Tables 2, 3. The three cases in the present work differ fundamentally from all previously published PWS dual- diagnosis reports, as they describe maternally inherited X-linked hemizygous variants rather than autosomal recessive homozygous mutations or sex chromosome aneuploidy. Full case summaries of retrieved literature are listed in Tables 2, 3.

**Table 2:** Summary of the genetic characteristics of PWS combined with sex chromosome disorders.

Literature	Gender	Genotype of PWS	Sex chromosome disorders	
			Karyotype	Disease
Trent, (17)	Male	Del(15q) pat	47, XXY	Klinefelter Syndrome
Rego, (6)	Male	Del(15q11q13) pat	47, XXY, del (15) (q11q13)	Klinefelter Syndrome
Butler, (7)	Male	UPhD(15) mat	47, XXY (origin of X chromosome: paternal nondisjunction)	Klinefelter Syndrome
Butler, (7)	Female	UPhD(15) mat	47, XXX (57%); 46, XX (43%) (origin of X chromosome: maternal meiosis II nondisjunction)	Trisomy X Syndrome
Devriendt, (18)	Female	UPhD(15) mat	47, XXX; (peripheral WBC) 47, XX [15]/ 47, XX, +15[16] (Primary skin fibroblast)	Trisomy X Syndrome
Geffroy, (15)	Male	Del(15q11q13) pat	47, XXY (origin of X chromosome: maternal nondisjunction)	Klinefelter Syndrome
Honma, (19)	Male	UPhD(15) mat	47, XYY	47, XYY syndrome
Odent, (20)	Male	Del(15q11q13) pat	47, XYY	47, XYY syndrome
Nowaczyk, (16)	Male	Del (15q11.2q13) pat	47, XXY, del (15) (q11.2q13) (origin of X chromosome: maternal nondisjunction)	Klinefelter Syndrome
Schneider, (21)	Male	Del (15q11.2) pat	47, XXY	Klinefelter Syndrome
Monzavi, (22)	Female	Del(15q11q13) pat	45, X, del (15) (q11q13)/47, XXX,del(15)(q11q13)	Turner syndrome
Carvalho, (23)	Male	Del (15q11q13) pat	47, XXY	Klinefelter Syndrome
Verhoeven, (8)	Male	UPiD(15q11q13 & 15q24q25) mat	47, XXY	Klinefelter Syndrome
Zeesman, (14)	Female	UPhD(15q11q14 & 15q25.3q26.3) mat Mixed UPiD & UPhD(15q14q25) mat	46, X, i(X) (q10)^a^	Turner syndrome

UPD, uniparental disomies; UPiD, uniparental isodisomy; UPhD, uniparental heterodisomy.

a Combining homozygous mutation in the HEXA gene.

**Table 3:** Summary of the genetic characteristics of PWS combined with other genetic disorders.

Literature	Gender	Genotype of PWS	Comorbid genetic disorders		
			Gene/+ Karyotype (Variation, Amino acid)	Inheritance, Source of variant	Disease
A. Autosomal chromosomal/genomic disorders (3 cases)					
Zarate, (24)	Female	Del (15q11.2q13.1) pat	47, XX, +21	Isolated cases	Down syndrome
Okamoto, (25)	Male	Del (15q11.2) pat	NSD1: del (5q35.2q35.3) pat, which led to haploinsufficiency of the gene,	AD, microdeletion of chromosome 5q35	Sotos syndrome
Zou, (9)	Male	UPD (15) mat	22q11.21 (18919528-21460595) x1	AD, 2.54 Mb deletion of chromosome 22q11.21	DiGeorge Syndrome
B. Autosomal recessive disorders unmasked by maternal UPD of chromosome 15 (9 cases)					
Woodage, (26)	Male	UPD (15) mat	Gene telomeric to D15S95 (15q25)	AR, homozygous, maternal	Bloom syndrome
Zeesman, (14)	Female	UPhD (15q11q14 & 15q25.3q26.3) mat Mixed UPiD & UPhD (15q14q25) mat	HEXA: 15q23, c.1274_1277 dupTATC (p.Ser426fs), NM_000520.6^a^	AR, homozygous, maternal	Tay–Sachs disease
Regier, (27)	Male	UPD (15q11.2q23.2) mat	HEXA: 15q23, c.1073 + 1G >A, NM_000520.6	AR, homozygous, maternal	Tay–Sachs disease
Karger, (31)	Male	UPD (15q11q22.2 & 15q26.1q26.3) mat	STRC and CATSPER2: 15q15.3 (43895633-43951301, 55.7 kb) deletion, which included these 2 genes,	AR, homozygous, maternal	Deafness–Infertility syndrome
Polubothu, (10)	Female	UPD (15) mat	CERS3: 15q26.3, c.716A >T (p.Asp239Val), NM_001378789.1	AR, homozygous, maternal	Autosomal recessive congenital ichthyosis
Muthusamy, (11)	Female	Mixed UPiD & UPhD(15q25.3q26.3)mat	CERS3: 15q26.3, c.23G >A (p.Trp8^a^), NM_001378789.1	AR, homozygous, maternal	Autosomal recessive congenital ichthyosis
Kunta, (28)	Male	UPiD(15) mat	SPG11: 15q21.1, c.733_734del (p.Met245fs), NM_025137.4	AR, homozygous, maternal	Hereditary spastic paraplegia type 11
Zhou, (29)	Male	UPiD(15q11.2q21.3) mat	IVD: 15q15.1, c.1006T >C (p.Cys336Arg), NM_002225.5	AR, homozygous, maternal	Isovaleric acidemia
Cuk, (30)	Female	UPiD(15q11.2q22.2) mat UPhD(15q22.2q26.3) mat	CAPN3: 15q15.1, c.550del (p.Thr184fs), NM_000070.3	AR, homozygous, maternal	CAPN3-associated calpainopathy
C. Other monogenic disorders unrelated to chromosome 15 UPD (3 cases)					
Jehee, (32)	Female, 2 cases of monozygotic twins	Del (15q11.2q13.1) pat	TCF4: 18q21.2, c.145 + 1G >A, NM_001083962.2	AD, heterozygous, maternal (the mosaic mother)	Pitt-Hopkins syndrome
Wu, (33)	Male	UPD (15) mat/Imprint center defects	ATP8A2: 13q12.3, c.187C >G (p.P63A), c.2138T >C (p.I713T), NM_016529.6	AR, compound heterozygous, paternal and maternal origin, respectively	Cerebellar ataxia, mental retardation, and disequilibrium syndrome type 4
D. Present case series (3 cases)					
Our cases	Male	UPD (15) mat	G6PD, Xq28, c.152C >T (p.Thr51Ile), NM_001360016.2	X-linked, hemizygous, maternal	Glucose 6-phosphatase deficiency
Our cases	Male	Mixed UPiD & UPhD(15)mat	G6PD, Xq28, c.1388G >A (p.Arg463His), NM_001360016.2	X-linked, hemizygous, maternal	Glucose 6-phosphatase deficiency
Our cases	Male	UPD (15) mat	G6PD, Xq28, c.1388G >A (p.Arg463His), NM_001360016.2	X-linked, hemizygous, maternal	Glucose 6-phosphatase deficiency

UPD, uniparental disomies; UPiD, uniparental isodisomy; UPhD, uniparental heterodisomy; AR, autosomal recessive; AD, autosomal dominant.

a Combined with i(X)(q10).

## Discussion

### Characteristics of PWS combined with sex chromosome abnormalities

Clinical descriptions of PWS superimposed on sex chromosome aneuploidy have accumulated over nearly seven decades (6–8, 17). Among the 14 collated cases, paternal deletion and maternal UPD PWS subtypes are evenly distributed (8:6). Extra X chromosomes in PWS-Klinefelter dual diagnosis typically originate from maternal meiosis I nondisjunction (15, 16), while one cohort documented an extra X derived from paternal gametogenic separation error (7). Trisomy X cases mostly acquired the supernumerary X chromosome via maternal meiosis II nondisjunction (7). At present, no consistent mechanistic linkage exists between chromosome 15 imprinting defects and sex chromosome segregation errors, regardless of paternal deletion, UPhD, UPiD, or mosaicism status (7). The uniform predominance of classic PWS phenotype across all dual-diagnosis patients may be explained by the more severe developmental injury induced by loss of paternal imprinted gene expression relative to X/Y chromosomal gene dosage imbalance (7). Most available data derive from studies published approximately 20 years ago, requiring additional contemporary sequencing evidence for validation.

### Phenotypic superimposition patterns of PWS combined with autosomal genetic disorders

Paternal deletion PWS concurrent with autosomal dominant disorders consistently exhibits blended phenotypes from both diseases (24, 25, 32). A 4-year-old girl with paternal deletion PWS and trisomy 21 displayed exacerbated congenital heart defects alongside canonical PWS hypotonia and feeding failure (24). A teenage boy with PWS and NSD1-deletion Sotos syndrome presented with atypical PWS frontal bossing, epilepsy, and hydronephrosis, plus aggravated hypotonia, intellectual disability, and scoliosis (25). Monozygotic twin females with paternal deletion PWS and TCF4-related Pitt–Hopkins syndrome demonstrated profound global neurodevelopmental delay, restricted vocabulary, and characteristic cerebral MRI ventricular dilatation and hippocampal hypoplasia (32). These observations suggest paternal imprinted gene loss exerts strong epistatic effects comparable to autosomal dominant pathogenic alleles under Mendelian inheritance rules (32).

### Nearly all autosomal recessive comorbidities arise in maternal UPD PWS patients

The concept of uniparental disomy was first introduced by Engel in 1980 (36), and its molecular mechanisms and clinical implications have been subsequently elaborated (37, 38), and incorporated into international best practice guidelines for PWS/AS molecular analysis (10, 11, 14, 26–31, 39). Maternal uniparental disomy provides two identical maternal copies of chromosome 15; if the mother carries a recessive pathogenic variant on chromosome 15, the child becomes homozygous and manifests the corresponding autosomal recessive disease (34). Several reported children with PWS plus autosomal recessive conditions lacked atypical clinical markers at initial diagnosis, likely due to young age, with recessive disease phenotypes emerging progressively with age (9, 29, 33). All identified variants in published cohorts were confirmed pathogenic across multiple public databases and functional literature.

### Clinical and genetic features of the current G6PD dual-diagnosis cohort

All three male infants demonstrated complete canonical PWS phenotypes without any clinical stigmata of G6PD deficiency, a phenotypic dissociation attributable to X-linked transmission patterns of the G6PD locus (12, 34). Aggregate analysis of prior maternal UPD PWS comorbidity series demonstrates that developmental dysregulation from chromosome 15 imprinting loss dominates clinical presentation over concurrent monogenic disease phenotypes (7, 24, 26). We hypothesize that embryonic loss of paternal imprinted gene function generates more profound developmental disturbance than autosomal biallelic variant imbalance or X-linked hemizygous mutation effects. A limitation of this study is the absence of refined quantitative G6PD enzyme activity testing in the cohort. Prior literature notes that female maternal UPD PWS patients may develop symptomatic X-linked disorders secondary to skewed X-chromosome inactivation (XCI) (34, 35). In contrast, all three subjects in this report are male hemizygous carriers inheriting a single mutant X chromosome from their mothers. It remains unclear whether this co-occurrence represents random chance or carries underlying genetic predisposition, requiring accumulation of additional analogous cases for mechanistic clarification.

### Clinical implications for expanded genetic testing in maternal UPD-PWS

This case series documents three novel observations of maternal UPD PWS concurrent with X-linked G6PD deficiency, supplementing rare dual-diagnosis data absent from prior literature. However, the extremely limited sample size of three patients prevents generalized conclusions regarding population-level genetic and clinical patterns, necessitating multi-center collaborative validation. Clinical practice has documented reverse diagnostic sequences where Down syndrome is initially suspected, with subsequent PWS methylation testing confirming paternal deletion (24). Standard karyotyping alone cannot detect single-gene variants hidden in maternal UPD patients. For individuals definitively diagnosed with maternal UPD via MS-MLPA and STR, trio-WES or trio-WGS should be prioritized when clinically feasible to screen maternally derived pathogenic variants on both chromosome 15 and the X chromosome, preventing missed dual genetic diagnoses and predicting risk of severe atypical composite phenotypes (34, 35). Expanded exome/genome sequencing retains critical diagnostic utility for maternal UPD-PWS patients even when no atypical clinical manifestations are apparent. Conclusions: This case series reports, for the first time to our knowledge, three clinical instances of maternal UPD-type PWS combined with X-linked hemizygous G6PD pathogenic variants, enriching the documented spectrum of PWS concurrent independent hereditary disorders and providing a new reference for multi-gene screening of imprinting disorders. All patients exhibited exclusive classic PWS phenotypes without any clinical manifestations attributable to G6PD deficiency. Clinicians managing maternal UPD-PWS patients should consider expanded genetic testing including trio- WES or trio-WGS where resources permit, to screen for hidden maternally transmitted multi-locus pathogenic variants, even in the absence of atypical physical signs. Owing to the small number of enrolled subjects in this work, further clinical data accumulation is required to fully delineate the genetic mechanisms and long-term phenotypic evolution of this combined disease entity.

## Data Availability

The datasets presented in this study can be found in online repositories. The names of the repository/repositories and accession number(s) can be found below: https://www.ncbi.nlm.nih.gov/snp/, rs2148332084, https://www.ncbi.nlm.nih.gov/dbvar/, SCV003925758, https://www.ncbi.nlm.nih.gov/dbvar/, SCV002599163.
